# Supplementary material for: Human-like monocular depth biases in deep neural networks
Source: PLoS Comput Biol. 2025 Aug 19;21(8):e1013020. doi: 10.1371/journal.pcbi.1013020 (PMC12380331; doi:10.1371/journal.pcbi.1013020)
Supplement: S1 Text — (PDF) [file pcbi.1013020.s001.pdf]

Appendix

Additional analysis for original participant data

To assess whether random half-splitting affected our findings, we compared the partial correlation between DNNs and human depth judgments before and after random half-splitting. Using the post-screening depth judgments, we computed the Pearson partial correlation between each participant's available judgments and each DNN's outputs, then averaged these correlations and obtained 95% confidence intervals. Although random half-splitting increased the overall similarity values, the underlying correlation structure and the ranking of DNNs by similarity remained essentially unchanged (Figs 4B, 5B, and S4). When we evaluated changes in the human similarity rankings with Spearman rank correlation, we observed very high agreement for both the absolute data (0.99, 95%CI $[0.99, 1.0]$) and scale-recovered data (0.96 95%CI $[0.93, 0.97]$). These findings indicate that random half-splitting can complete missing data and reduce noise without substantially altering the human similarity rankings of the DNNs.

Additional analysis for supplemental dataset

In addition to the main dataset, we investigated how depth judgments by humans and DNNs behave in data derived from a different task (supplemental dataset). This Appendix provides a concise overview of the methods and key findings from this supplemental analysis.

The supplemental dataset was collected to assess human judgments of relative depth ratio and depth order between pairs of points, and to compare their error patterns with those of the DNN model outputs. For the relative depth judgments ('relative'), we employed the Pearson partial correlation of signed relative values, quantified as $\log_{10}(z_{\text{ans}}^{(2)}/z_{\text{ans}}^{(1)})$, to measure error pattern similarity. For the two-alternative forced-choice (2AFC) data ('2AFC'), we calculated error consistency using the same approach as applied to the pairwise data derived from the main dataset (for details, please see the section titled: Disentangling metric and ordinal error components by ordinal-level analysis).

S5A Fig presents scatter plots comparing model accuracy, as quantified by scale-shift invariant RMSE, against similarity to human judgments derived from the supplemental dataset ('relative' and '2afc'). In each plot, the pink horizontal line indicates the inter-individual similarity in human depth judgments between participant judgments. Marker colors correspond to the type of training dataset used, and marker shapes denote the specific training strategies employed. Error bars along the vertical axis represent the 95% confidence intervals for partial correlation and error consistency.

Our analysis revealed that the four measures derived from two different tasks exhibited a highly similar data structure. To evaluate the consistency of model rankings based on human similarity across these measures, we computed the Spearman rank correlations of model rankings across all pairwise combinations of the four distinct measures (S5B Fig). This analysis demonstrated strong positive correlations between the measures derived from the supplemental dataset ('relative' and '2AFC') and those from main dataset ('raw' and 'rank'). These findings suggest that specific depth judgment paradigm (absolute vs. relative) has minimal influence on the observed patterns of human and DNN depth judgments, and that our primary findings based on absolute depth judgments are robust and generalizable to relative depth judgment tasks commonly employed in previous studies.
