# Supplementary figures and images for: Human-like monocular depth biases in deep neural networks
Source: PLoS Comput Biol. 2025 Aug 19;21(8):e1013020. doi: 10.1371/journal.pcbi.1013020 (PMC12380331; doi:10.1371/journal.pcbi.1013020)

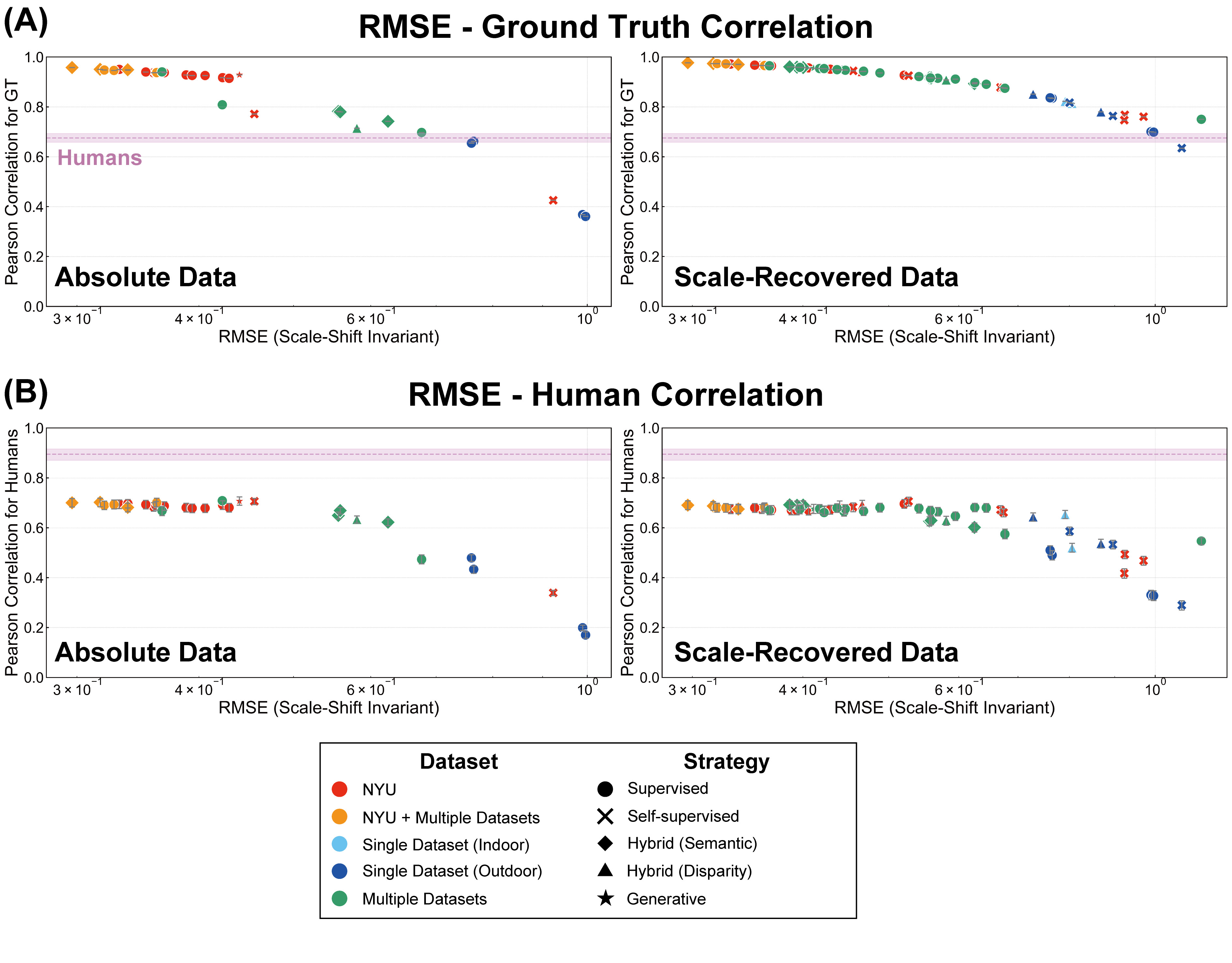

Supplement: S1 Fig — (A) Scatter plot of scale-shift invariant RMSE versus the Pearson correlation between DNN output and ground truth. (B) Scatter plot of scale-shift invariant RMSE versus the Pearson correlation between DNN outputs and human data. (TIF) [file pcbi.1013020.s002.tif]

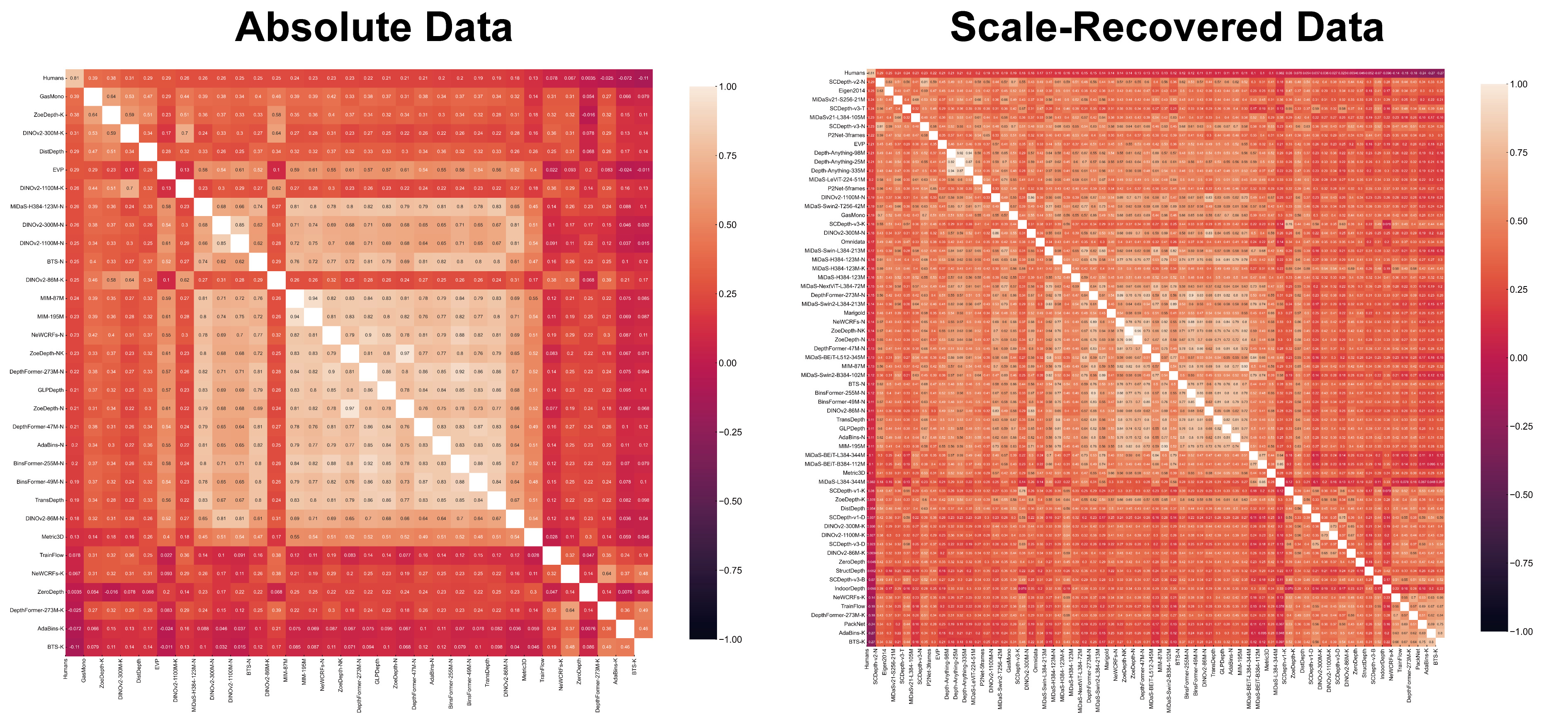

Supplement: S2 Fig — (Left) Heatmap for the absolute data, showing Pearson partial correlations among humans and 31 absolute-value DNNs. (Right) Heatmap for the scale-recovered data, showing Pearson partial correlations among humans and 64 scale-recovered DNNs. In both panels, the top row and leftmost column correspond to human-DNN similarities; DNNs are ordered by descending human similarity. (TIF) [file pcbi.1013020.s003.tif]

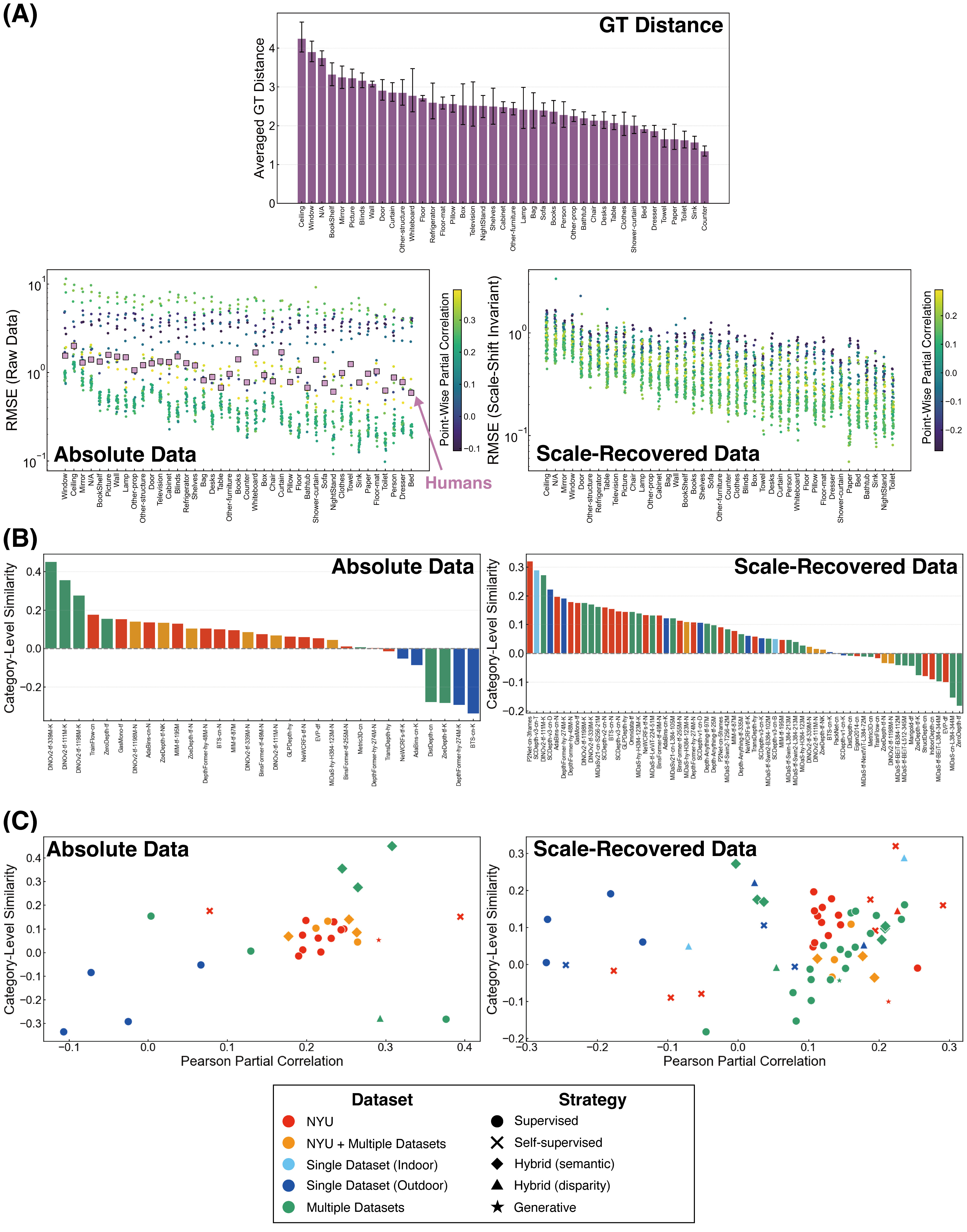

Supplement: S3 Fig — (A) Per-category RMSE analysis. The top plot shows the average ground truth distance for each semantic category. The plots below show the per-category RMSE for humans (purple squares) and DNNs (dots) for both absolute and scale-recovered data. DNN models are color-coded by their overall similarity to human judgments (measured by point-wise partial correlation). (B) Category-level human similarity for each DNN model. This metric measures the Pearson partial correlation between human and DNN per-category error patterns (from panel A) while controlling for per-category ground truth distance. (C) Relationships between category-level human similarity (from panel B) with overall point-wise human similarity. (TIF) [file pcbi.1013020.s004.tif]

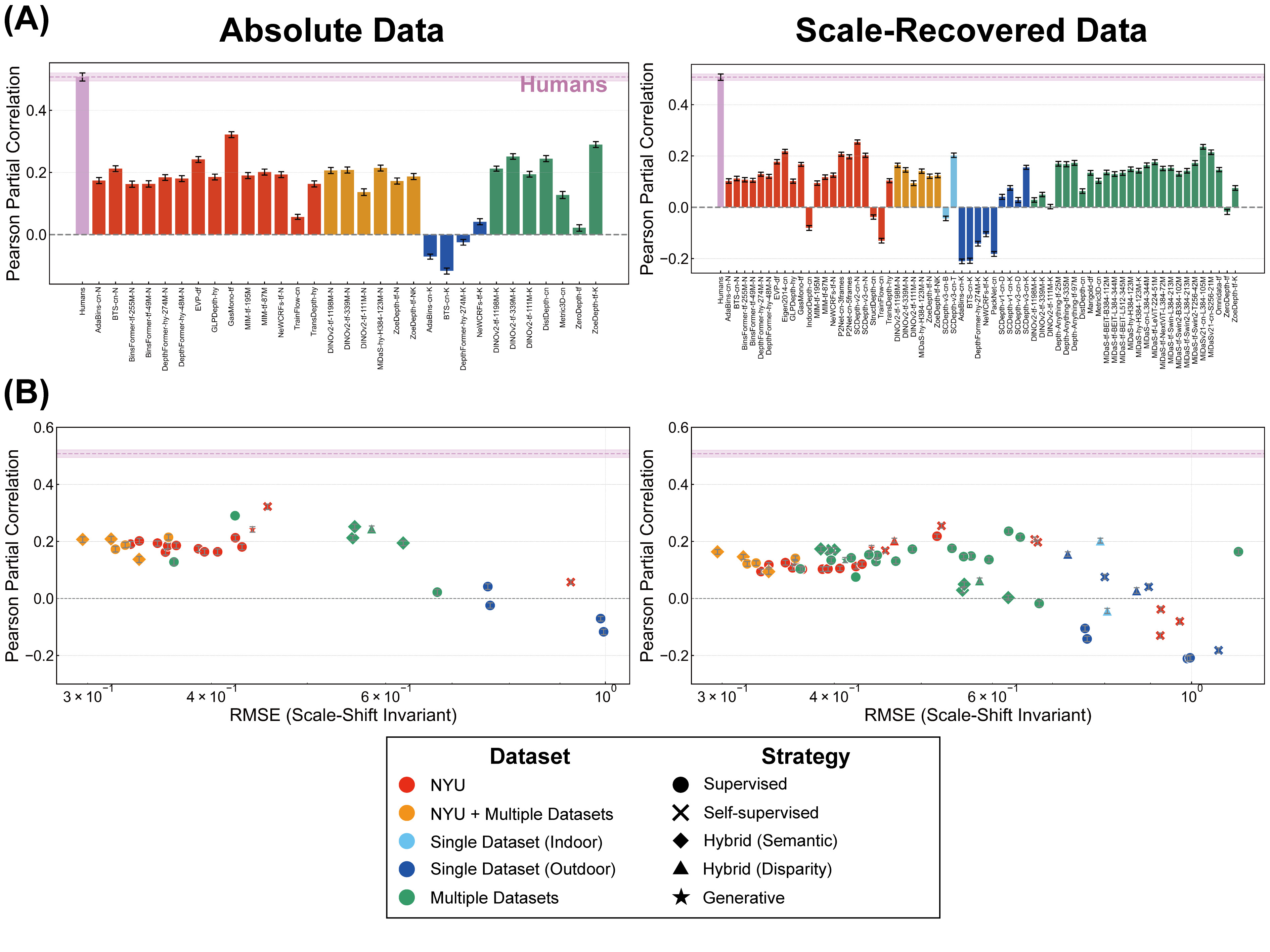

Supplement: S4 Fig — (A) Similarity between humans and DNNs based on Pearson partial correlations. (B) Scatter plot showing the relationship between scale-shift invariant RMSE and human similarity. For both absolute (left) and scale-recovered (right) analyses, the inter-human partial correlations were calculated from absolute data, serving as a reference benchmark for human-level consistency. These results are highly consistent with those derived from the half-split procedure (Figs 4B and 5B). (TIF) [file pcbi.1013020.s005.tif]

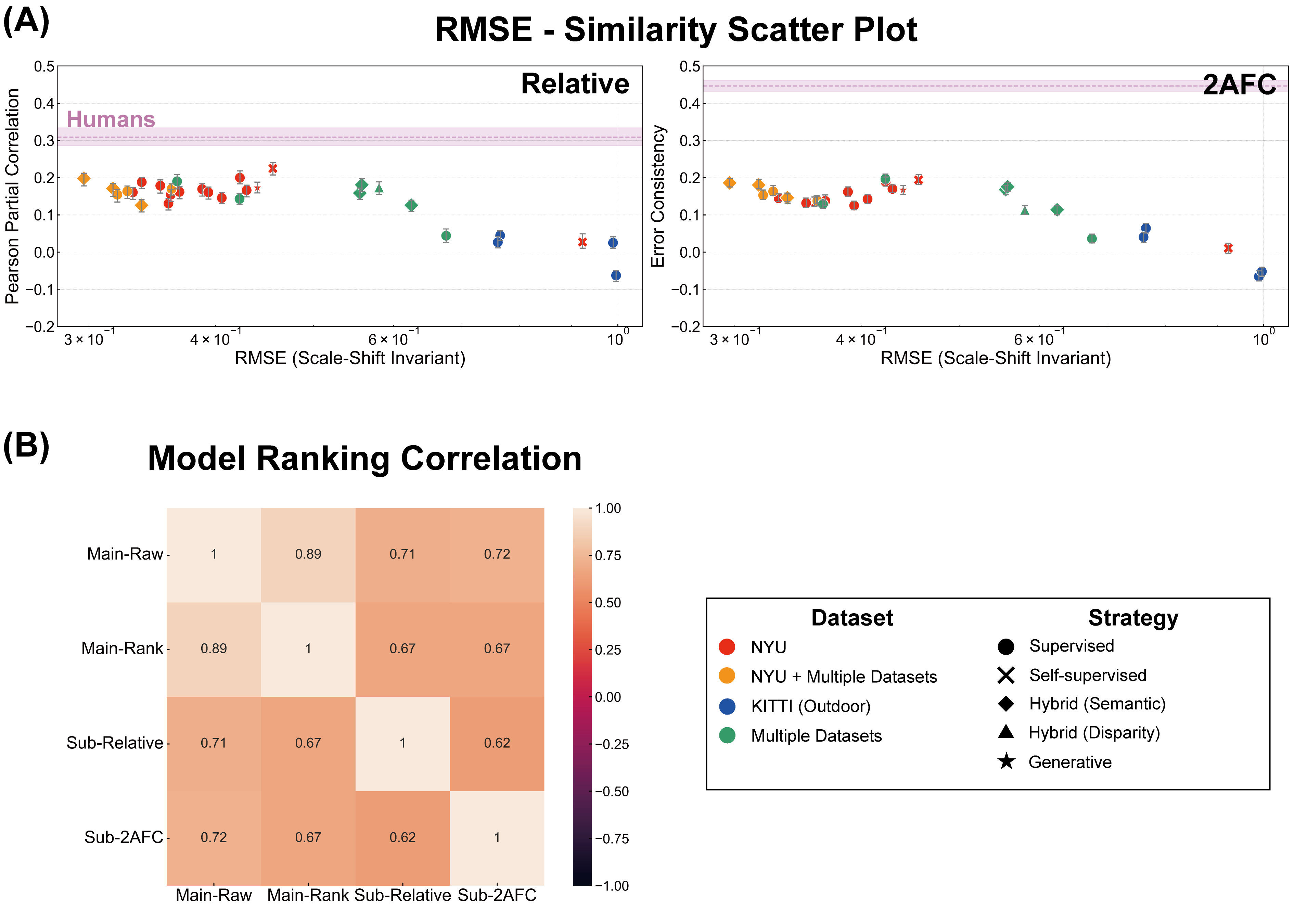

Supplement: S5 Fig — (A) Scatter plots illustrating the relationship between scale-shift invariant RMSE and human similarity by two measures. Marker colors indicate the type of training datasets, while marker shapes represent the training strategy used. (B) Spearman correlation coefficients for model rankings based on human similarity across four distinct measures. We analyze both main and supplemental data in this graph using original depth judgments instead of random half-split data. (TIF) [file pcbi.1013020.s006.tif]

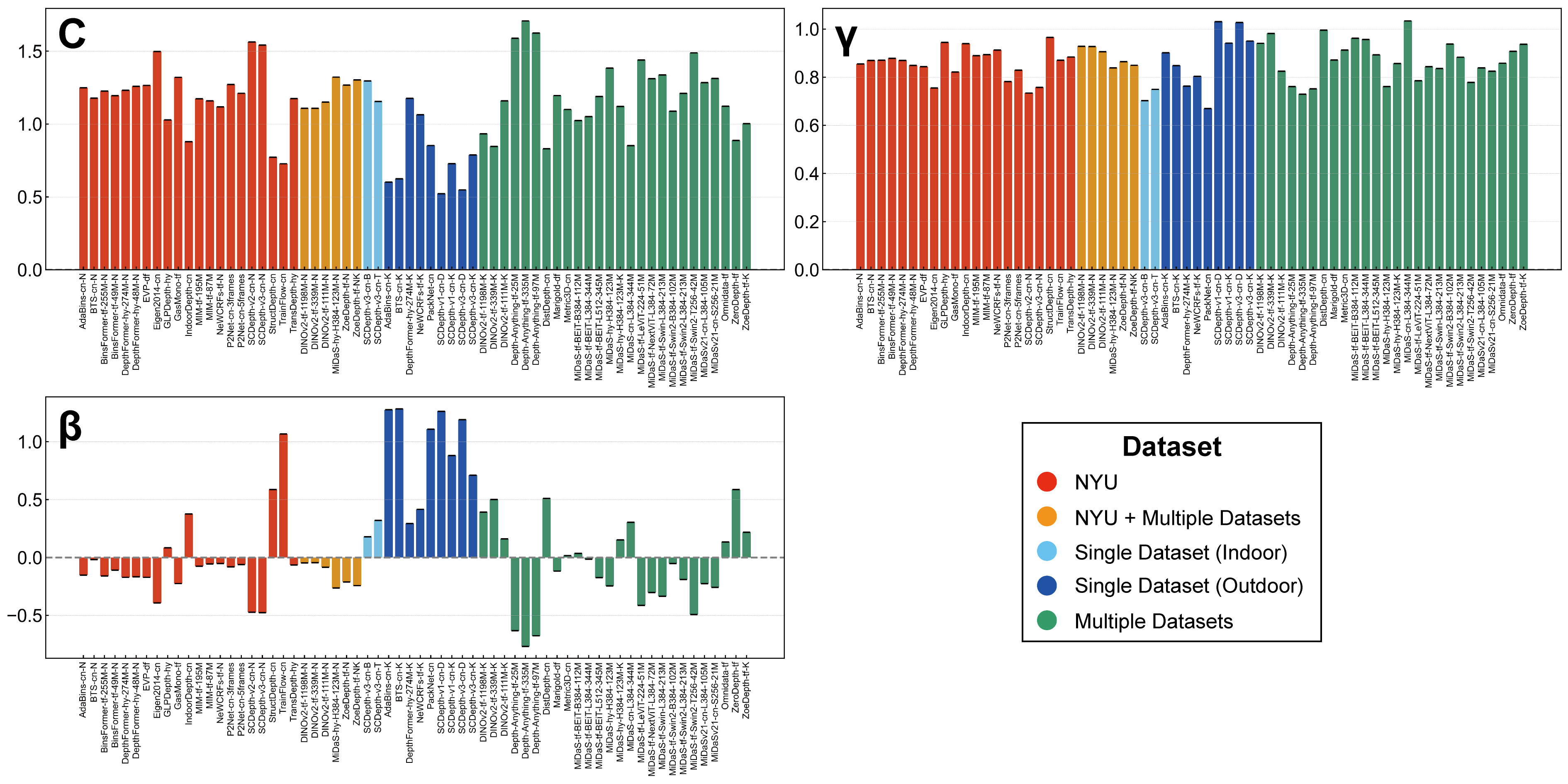

Supplement: S6 Fig — The figure consists of three subplots: scale component (C), exponent component (γ), and shift component (β). (TIF) [file pcbi.1013020.s007.tif]

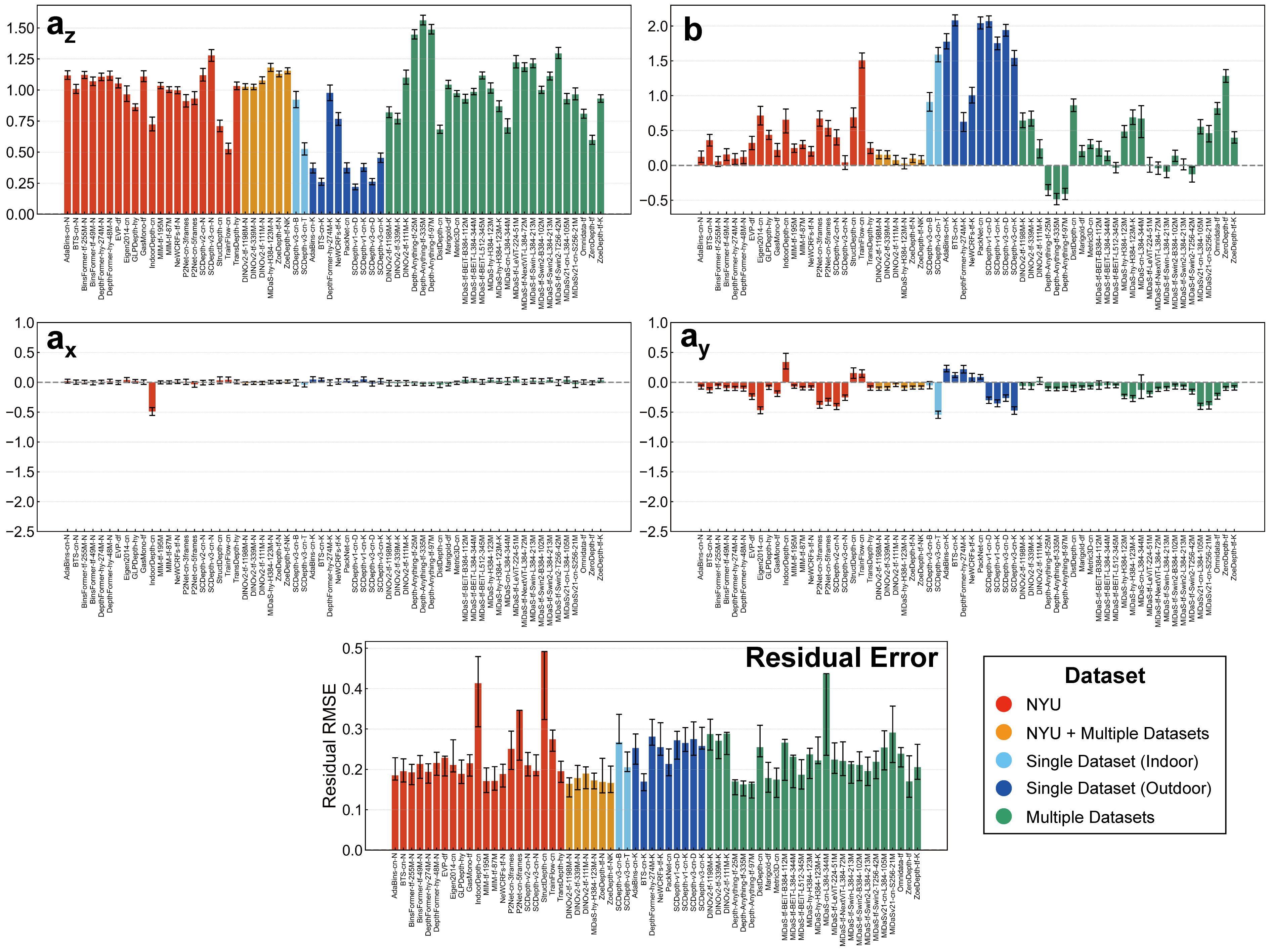

Supplement: S7 Fig — The figure comprises five subplots: scale component (az), shift component (b), horizontal shear component (ax), vertical shear component (ay), and RMSE for residual error. Error bars represent the 95% confidence intervals derived from random half-split human data. (TIF) [file pcbi.1013020.s008.tif]
