# Supplementary material for: Human-like monocular depth biases in deep neural networks
Source: PLoS Comput Biol. 2025 Aug 19;21(8):e1013020. doi: 10.1371/journal.pcbi.1013020 (PMC12380331; doi:10.1371/journal.pcbi.1013020)
Supplement: Attachment — Submitted filename: response_to_reviewers.pdf [file pcbi.1013020.s010.pdf]

Authors' response to the reviewer's comments

This document presents our replies to the reviewers' feedback. The text colors used in this document indicate the following:

- Gray: Reviewer's comments that do not require a response
- Red: Reviewer's comments requiring a response
- Blue: Authors' responses and notes about changes
- Black: Authors' revised and added sentences; Page numbers refer to the revised manuscript (manuscript.pdf)
- **Green bold: Serial numbers of comments and responses**

This document contains the following:

1. Responses to Reviewer #1
2. Responses to Reviewer #2
3. Additional Responses to Reviewers
4. Responses to Editors

1. Responses to Reviewer #1

This study investigates how humans and deep neural networks (DNNs) perceive depth from 2D images, revealing that both exhibit systematic distortions such as depth compression and vertical biases. By analyzing a novel human-annotated dataset using affine decomposition, the authors show that more accurate DNNs partially mirror human error patterns, suggesting these biases may reflect ecologically efficient strategies. The work highlights the value of examining error structures beyond raw accuracy to better understand human and model depth perception.
The paper is well-written, well-organized, and easy to follow. The use of absolute depth judgments sets it apart from existing studies, and the experimental design is thoughtful and adds meaningful value to the community. The analysis of depth biases in both humans and models is particularly insightful.
However, I have several concerns regarding the evaluation metrics, experimental design, and missing comparisons with recent state-of-the-art models. Addressing these issues would significantly strengthen the paper.

We deeply appreciate the reviewer's constructive suggestions, which we believe enhance the rigorousness, significance, and value of our paper. The following is our responses to the points raised by Reviewer #1.

#R1_1 Error Patterns and Adversarial Stimuli ---

1. If the authors can identify systematic error patterns in both human and model predictions, they could potentially design adversarial stimuli that exploit these patterns to elicit mistakes. This direction would be a valuable avenue for future work and should be discussed.

We agree that identifying systematic error patterns opens up a fascinating avenue for future work: designing adversarial stimuli to probe the underlying mechanisms of depth perception in both humans and DNNs.
As the reviewer suggested, one could leverage our findings on image-specific affine distortions by generating controlled stimuli (e.g., using CGI) that are specifically designed to elicit these specific types of misperceptions in DNNs. By then testing human perception with these same "model-adversarial" images, we could more rigorously test whether the computational principles and failure modes of DNNs align with those of human vision. This approach would allow us to move beyond correlational analyses toward a more causal understanding of the similarities and differences between biological and artificial visual systems.

We have added a paragraph to our Discussion section to explicitly mention this promising future direction.

(Discussion, p.21, ll.869-875)
A particularly exciting future direction lies in uncovering the origins of the image-specific affine biases we observed. A computational search through scene parameters (e.g., geometry, textures, and camera properties) could identify the specific visual configurations that elicit affine errors in DNNs. Subsequently testing humans with these machine-generated "illusions" would provide a deep and causal test of human-AI alignment, revealing whether the human visual system shares the same vulnerabilities.

#R1_2 Human-Human Consistency v.s. Model-Model Consistency---

2. What is the depth estimation consistency across different human observers? Quantifying this would provide a strong baseline for evaluating human-machine agreement. Similarly, consistency across different machine models should also be reported.

We agree that quantifying human-human and machine-machine consistency provides crucial baselines for evaluating human-machine agreement. In response, we have performed two additional analyses to strengthen the manuscript.

(a) Depth estimation consistency across different human observers
The reviewer correctly highlights the importance of quantifying human-human consistency as a baseline. In our original manuscript, we reported this consistency using a random half-split procedure, which yielded a Pearson partial correlation of 0.808 (95% CI [0.766, 0.845]). We believe this "pseudo-participant" approach is well-suited for our study because (1) it allows us to evaluate consistency across the entire set of 654 images, overcoming the limitation that each participant only saw a subset, and (2) it effectively reduces individual response noise, providing a clearer measure of the shared, systematic components of human depth perception.

To further address the reviewer's point and strengthen our analysis, we have made the following revisions:

Clarified the Rationale in the Main Text: We have revised the text in the Results section to more explicitly state that this human-human correlation serves as the "human-level baseline" or "ceiling" for evaluating the performance of the DNNs. This makes the purpose of this analysis clearer.

(Results > Dataset collection for human depth judgments, p.5, ll.158-168)
To establish a human-level baseline or ``ceiling" for evaluating the performance of the DNNs, we needed to quantify the consistency of depth judgments across human observers. In the experiment, each participant responded to only a subset of the 654 images. To reliably measure how humans agree with each other across the full dataset, we adopted a random half-split procedure. This involves creating two ``pseudo-participant'' datasets by randomly splitting and averaging the observers. This approach provides full data coverage and, by averaging out individual-specific response noise, allows for a more robust measurement of the error patterns that are systematically shared among human observers. By repeating this split 1,000 times, we obtained robust estimates and their 95% confidence intervals (CIs) for all subsequent human data analyses unless otherwise specified (see Materials and methods for detail).

Added a Robustness Check: To validate our half-split approach, we performed additional analyses on a subset of data where pairs of actual participants responded to the same images. These new analyses confirm that our main findings are robust and not an artifact of the half-splitting procedure.

Specifically, we performed two additional analyses:

(i) Raw Inter-Observer Consistency
We directly computed the depth judgments consistency between pairs of individual human observers on the raw data (i.e., before the half-split averaging). The result exhibited an average Pearson correlation of 0.669 (95%CI [0.659, 0.679]) and a partial correlation (controlling for ground truth) of 0.508 (95%CI [0.495, 0.521]). While these values are lower than those from our half-split data, which is expected due to the presence of individual response noise, they still indicate a strong, positive agreement among observers. This confirms that the shared systematic biases are present in the original data, and our half-split method effectively provides a less noisy estimate of this shared consistency. Crucially, even if we were to use this lower, more conservative value as the human-level baseline, our main conclusion remains unchanged: there is a significant gap between the human-level consistency and the human-DNN similarity observed for all tested models. Our half-split method simply provides a less noisy, and thus higher, estimate of this ceiling. We have added a brief summary of this finding to the main text (Results, p. 6).

(ii) Stability of Human-DNN Similarity Rankings
To ensure our half-split procedure did not distort the core findings of our human-DNN comparison, we compared the similarity rankings of the DNN models calculated using both the raw (pre-split) and the half-split data. The Spearman rank correlation between the two sets of rankings was exceptionally high for both absolute data (ρ = 0.99, 95%CI [0.99, 1.0], and scale-recovered data (ρ = 0.96 [0.93, 0.97]). This demonstrates that the use of random half-split is a promising method for analyzing the correlation structure between models and humans while reducing noise in human data without altering the fundamental structure of human-DNN similarity.

We have added these analyses and discussions to the manuscript (Results, p. 6 and p. 14) and included a new figure (S4 Fig.) in the Supporting Information to present these results visually. We are confident that these additions substantially strengthen the validity of our findings.

(Results > Human depth judgments exhibit a substantially shared error pattern, p.6, ll.194-198)
Furthermore, a similar analysis performed on the original raw data (i.e., before the half-split averaging) also revealed a strong positive correlation among observers: an average correlation of 0.669 (95%CI [0.659, 0.679]) and a partial correlation of 0.508 (95%CI [0.495, 0.521]), confirming that this high consistency is not an artifact of our procedure.

(Results > Human-DNN similarity improves with invariant accuracy measures, p.13, ll.524-528)

Notably, these conclusions are robust. We verified that the model similarity rankings remain highly consistent (Spearman's $\rho > 0.96$) even when using the original raw participant data, as opposed to the noise-reduced ``pseudo-participant" data generated by our half-split procedure (see Supporting Information, S1 Appendix and S4 Fig.).

(Supporting Information > S1 Appendix)
To assess whether random half-splitting affected our findings, we compared the DNN similarity results against those derived directly from the original, non-split participant data. Using the post-screening depth judgments, we computed the Pearson partial correlation between each participant's available judgments and each DNN's outputs, then averaged these correlations and obtained 95% confidence intervals. While the absolute similarity values were higher with the half-split data due to its noise-reduction effect, the human similarity ranking of DNNs remained essentially unchanged (Figures 4B, 5B, and S4 Fig). To confirm this, we compared the human similarity rankings from both methods with Spearman rank correlation and observed very high agreement for both the absolute (0.99, 95%CI [0.99, 1.0]) and scale-recovered data (0.96, 95%CI [0.93, 0.97]). This indicates that the random half-split procedure is a robust method that effectively reduces noise without altering the human similarity rankings of the DNNs.

(Supporting Information > S4 Fig)
Results of human-DNN similarity analysis using the individual participant’s data without half-split procedure. (A) Similarity between humans and DNNs based on Pearson partial correlations. (B) Scatter plot showing the relationship between scale-shift invariant RMSE and human similarity. These results are highly consistent with those derived from the half-split procedure (Figs. 4B and 5B). (TIF)

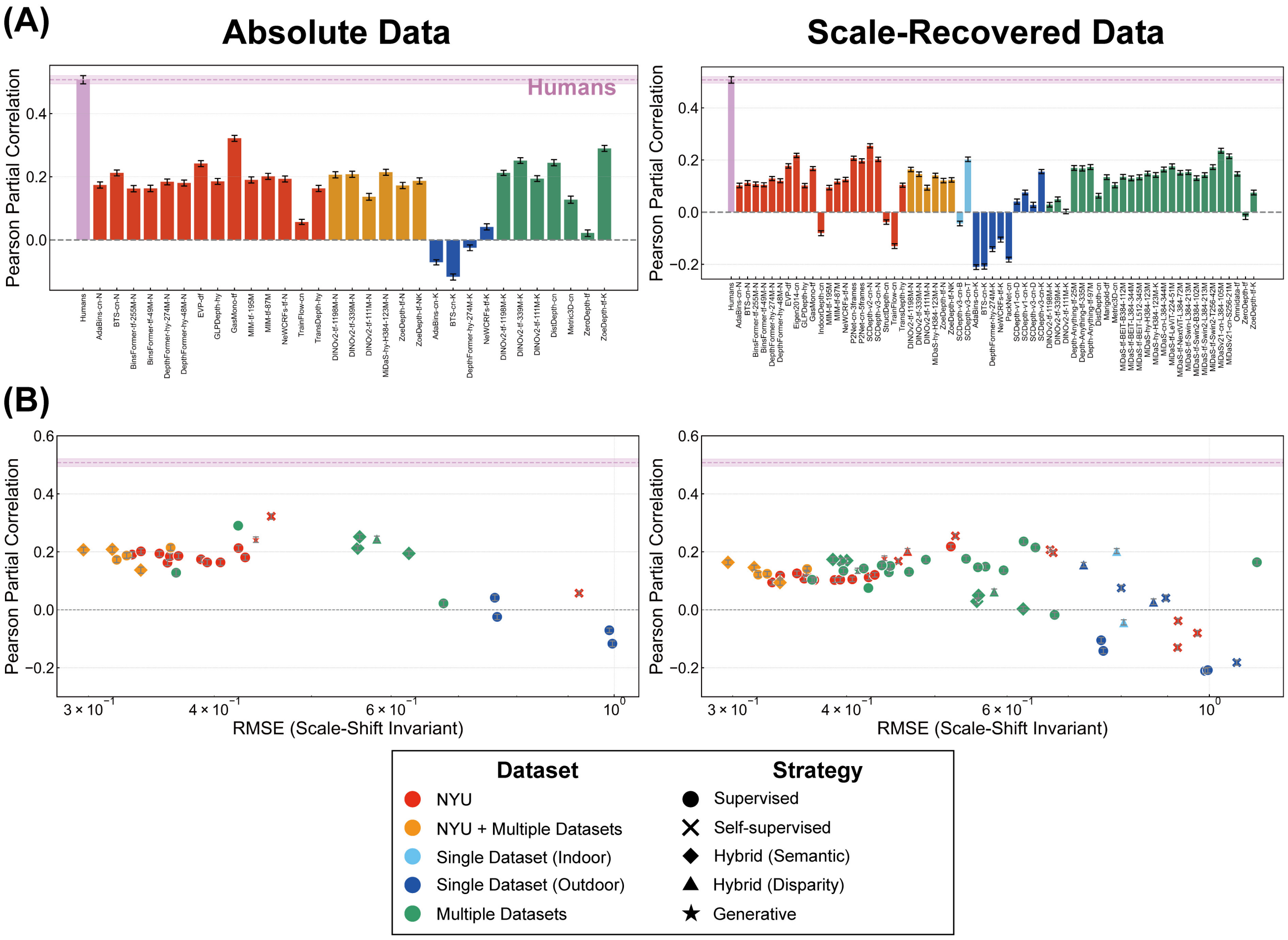

(b) Depth estimation consistency across different machine models
Following the reviewer’s suggestion, we analyzed the depth judgments consistency across different machine models. We computed the pairwise Pearson partial correlations among DNN models and visualized the results in a heatmap shown in S2 Fig. Our key finding is that the average DNN-DNN similarity is substantially higher than the

average human-DNN similarity for both absolute data (human-DNN similarity: 0.194, 95%CI [0.151, 0.238], DNN-DNN similarity: 0.416, 95%CI [0.392, 0.440]) and scale-recovered data (human-DNN similarity: 0.097, 95%CI [0.065, 0.129], DNN-DNN similarity: 0.435, 95%CI [0.429, 0.442]). This suggests that while different models share some error patterns with humans, they tend to converge on solutions that are more similar to each other than to human perception, forming a distinct "machine cluster" of error patterns. We have added a paragraph to the main text to report this finding and its implications, directly referencing a new supplementary figure, as follows:

(Results > DNN depth judgments partially share human error patterns, p.12, ll.455-469)
While the human-DNN comparison reveals a degree of shared bias, it also raises the question of how error patterns vary among DNNs themselves. To investigate this, we computed the Pearson partial correlation between all pairs of 31 absolute-value DNNs and separately among 64 scale-recovered DNNs, then visualized these results as heatmaps (S1 Fig). The heatmap reveals a rich internal structure within the DNN error patterns. Notably, there are visible clusters of high similarity, particularly among models trained on the same i.i.d. datasets (i.e., NYU Depth V2), while models trained only on o.o.d. data (i.e., KITTI) tend to show lower similarity with these clusters. Despite this internal variance, the overall trend is clear. On average, the DNN-DNN similarity is significantly higher than the average human-DNN similarity in both absolute data (human-DNN similarity: 0.194, 95%CI[0.151, 0.238], DNN-DNN similarity: 0.416, 95%CI [0.392, 0.440]) and scale-recovered data (human-DNN similarity: 0.097, 95%CI [0.065, 0.129], DNN-DNN similarity: 0.435, 95%CI [0.429, 0.442]). This indicates that while DNNs do share some error structure with humans, they share substantially more with other DNNs.

(Supporting Information > S2 Fig)
Error pattern similarity among humans and DNNs. (Left) Heatmap for the absolute data, showing Pearson partial correlations among humans and 31 absolute-value DNNs. (Right) Heatmap for the scale-recovered data, showing Pearson partial correlations among humans and 64 scale-recovered DNNs. In both panels, the top row and leftmost column correspond to human-DNN similarities; DNNs are ordered by descending human similarity.

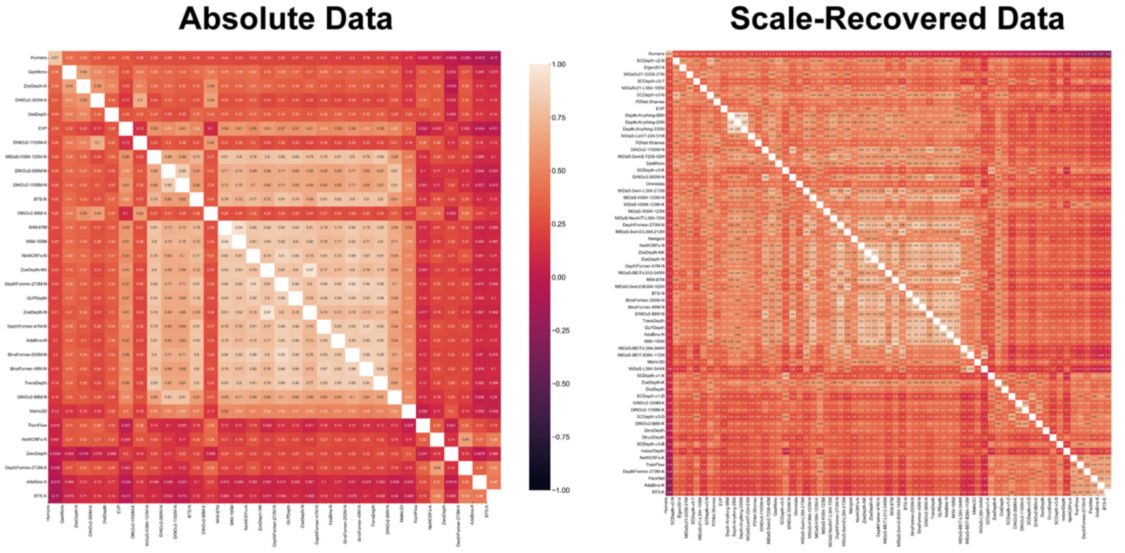

Furthermore, to ensure the entire manuscript is coherent with this more nuanced conclusion, we have carefully revised the Author Summary and the Discussion section. These revisions clarify that while more accurate DNNs show a tendency to exhibit certain human-like biases, they remain fundamentally within a solution space that is distinct from human vision.

(Author Summary)
Strikingly, the better the DNNs were at depth estimation, the more they also exhibited human-like biases. This suggests that these seemingly suboptimal human biases could in fact reflect efficient strategies for inferring 3D structure from ambiguous 2D inputs. However, we also found that the models' error patterns are more similar to each other than to humans, and they struggle to match human performance in judging the relative order of objects in depth, especially when we accounted for viewpoint distortions. We believe that our dataset and discovery of multiple error factors will drive further comparative studies between humans and DNNs, facilitating model evaluations that go

beyond simple accuracy to uncover how depth perception truly works---and how it might best be replicated in computational models.

(Discussion, p.20, ll.807-815)
However, it is important to note that despite these shared biases, a substantial gap remains. Human-human consistency (partial correlation ~ 0.8) still far exceeds the highest human-DNN similarity we observed (~0.4). Our analysis provides a crucial insight into this gap: DNNs, regardless of their performance, form a distinct cluster of error patterns, being systematically more similar to each other than to humans (S2 Fig).
This suggests a nuanced relationship: while increasing model accuracy may push DNNs towards the human strategy in certain aspects, the models are still largely constrained within a solution space that is fundamentally different from that of human vision. Indeed, ….

#R1_3 RMSE for Humans ---
3. Why is RMSE reported only for machine predictions? It would be informative to compute the same metric for human estimates to allow for a more direct and fair comparison.

The reason we do not report a scale-recovered RMSE for human estimates is that it would not be directly comparable to the RMSE for the DNNs, making such a comparison inappropriate for a fair evaluation. For the DNNs, the RMSE is calculated from their scale-recovered depth estimates. To obtain these, we fit their dense, pixel-wise output maps to the ground truth. This approach provides a stable and robust basis for ranking the performance across different models. For the human data, which is inherently sparse (8 points per image), applying the same process would be less ideal for this specific purpose of robust performance ranking. Our primary analysis of human-model similarity therefore compares the models' scale-recovered outputs to the raw, absolute depth judgments from humans, as stated in the manuscript: "*We derived metric-aligned pseudo-absolute depth data by linearly regressing each DNN's output depth map (for all 64 models) against the ground truth depth map for each image (see Materials and Methods for details). Then, we compared these scale-recovered DNN outputs to raw random half-split human judgments (i.e., pseudo-participants' data), keeping our human baseline in its original, absolute scale.* (p.11, ll.399-403)"

Consequently, a direct RMSE comparison would involve a metric based on scale-recovered data (for DNNs) and one based on raw, absolute data (for humans). This comparison would be inequitable; the DNN's RMSE is calculated after its outputs are optimally scaled to the ground truth, whereas the human data consists of raw judgments that retain their inherent systematic scaling biases. Since RMSE is highly sensitive to such overall scaling and offset, a direct comparison of these values derived from differently processed data would be misleading. To avoid any confusion for our readers, we have added a brief note to the manuscript to clarify why the human RMSE is not presented in the scale-recovered analyses."

(Results > DNN depth judgments partially share human error patterns, pp.11-12, ll.434-439)
… This expanded analysis revealed similar error tendencies related to their training datasets, mostly consistent with our initial findings. Note that the human RMSE is shown only in the left column, as a direct comparison with the models' scale-recovered RMSE would be misleading; the latter is calculated after optimal scaling to the ground truth, whereas the human data retains its inherent scaling biases.

When comparing error patterns using partial correlation, we consistently use the human-human correlation, calculated from raw judgments, as a reference benchmark. This applies to comparisons with both the models' absolute and scale-recovered data. We acknowledge the methodological asymmetry in the latter case; however, the comparison remains highly informative for assessing the similarity of error patterns, a measure less sensitive to absolute scaling biases than RMSE. To ensure full transparency, we have noted in the caption of Figure 4 that this benchmark is derived from raw data. This approach provides a crucial, albeit not perfectly equivalent, reference for human-level consistency across all relevant analyses.

(Fig 4)
Comparison of human and DNN error patterns. (A) Accuracy as measured by RMSE for raw error. (B) Similarity between humans and DNNs based on Pearson partial correlation. For both absolute (left) and scale-recovered (right) analyses, the inter-human partial correlations were calculated from absolute data, serving as a reference benchmark for human-level consistency.

Finally, it is worth noting that for other analyses where we compare the structural properties of errors (i.e., in our affine decomposition), we intentionally use only the 8 corresponding points for both humans and DNNs. This is because the goal there is to ensure a fair comparison of distortion patterns under identical, albeit sparse, conditions. However, for a global accuracy metric like RMSE, we believe relying on the most robust data available for each source and acknowledging the limits of their direct comparison is the most rigorous approach.

#R1_4, 1_5 Depth Comparison: Human vs Machine ---

4. In Figure 2, human depth estimates are compared to ground truth. Why isn't the same comparison made for machine predictions? Instead of comparing RMSE values indirectly, it would be more informative to directly compare machine-predicted depths to human estimates.
5. Building on point 4, what are the correlation values between machine-predicted depths and ground truth? Additionally, what is the correlation between machine estimates and human estimates?

In response to the reviewer's suggestion, we have performed the additional analyses comparing machine-predicted depths directly to both ground truth (GT) and human estimates. We have included these results in a new Supplemental Figure (S1 Fig). Our analysis shows that while top-performing DNNs have high correlations with both GT and human data, these simple correlation metrics are not sensitive enough to differentiate the nuanced, human-like error patterns between models, as performance tends to saturate.

For this reason, our main analysis focuses on the partial correlation of errors. This method allows us to specifically assess the similarity of systematic biases shared between humans and DNNs, beyond what can be explained by their shared high accuracy. We believe this provides a more stringent and meaningful measure of "human-likeness."

We have updated the manuscript to briefly mention these supplementary findings and to better motivate our choice of analytical methods.

(Results > Common biases in depth judgments among humans, p.6, ll.179-184)
To more precisely assess similarities in participants' error patterns, we computed a Pearson partial correlation between the split datasets while controlling for the ground truth. This approach allows us to distinguish whether the high consistency between observers arises simply because they are both accurately perceiving the true depth, or because they share a common pattern of systematic biases. Specifically…

(Results > DNN depth judgments partially share human error patterns, p.12, ll.440-448)
However, simple accuracy metrics like RMSE can be insufficient, as they often saturate for high-performing models. Similarly, raw correlations with both the ground truth and human judgments are also very high for these models, often saturating and thus obscuring nuanced differences in their error patterns (see S1 Fig). To provide a more sensitive measure of human-likeness, we therefore analyze the similarity of the error patterns themselves by computing the Pearson partial correlation, which controls for the ground truth. This is crucial for determining whether human and DNN judgments align merely due to their shared accuracy, or because they share genuine systematic biases.

(Supporting Information > S1 Fig)
Relationship between DNN accuracy and similarity scores: correlation with ground truth and human judgments. (A) Scatter plot of scale-shift invariant RMSE versus the Pearson correlation between DNN output and ground truth. (B) Scatter plot of scale-shift invariant RMSE versus the Pearson correlation between DNN outputs and human data.

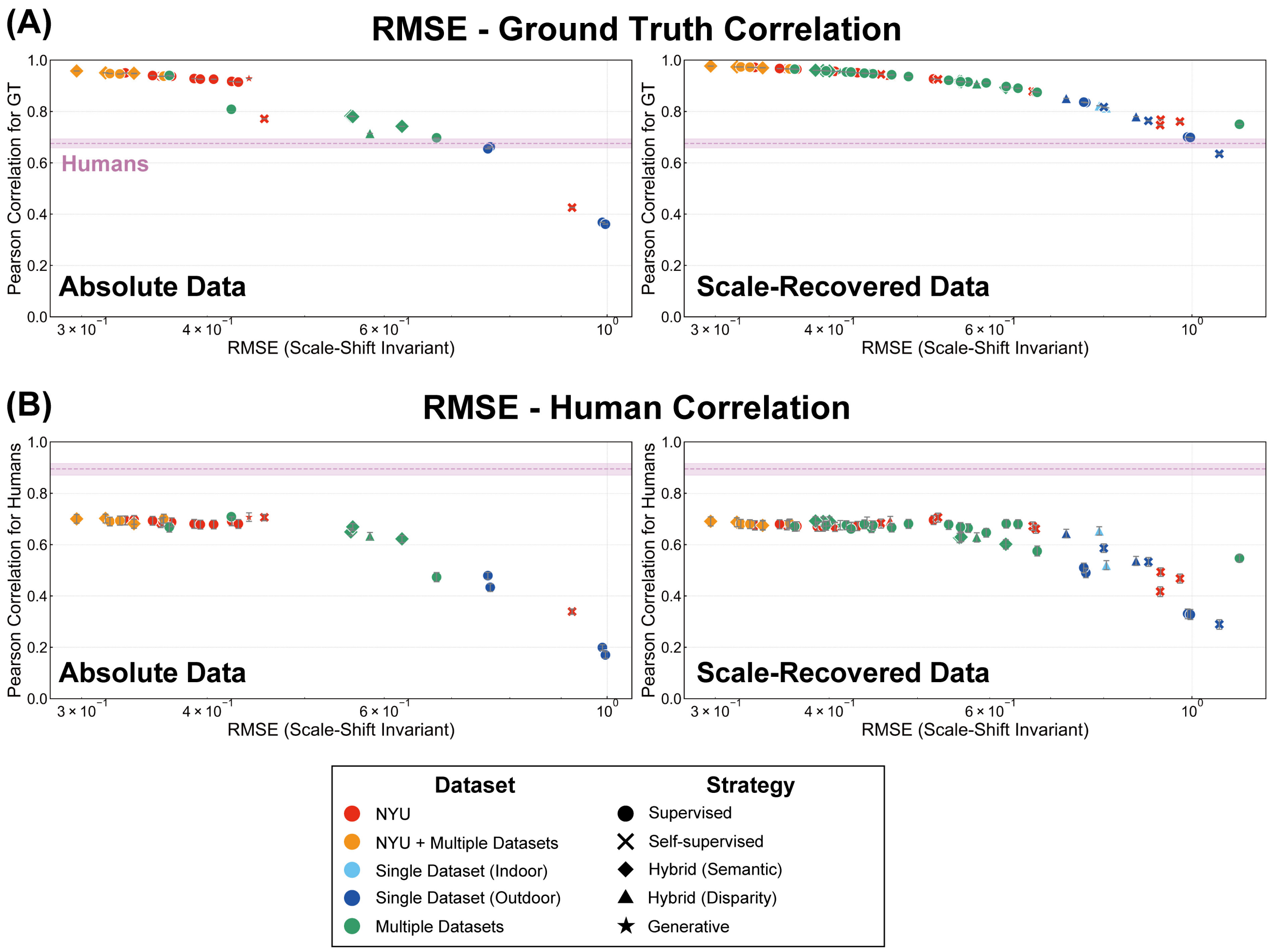

#R1_6 Figure Legends --

6. In Figures 9 and 11 (and possibly others), the light pink bars represent human performance. This should be clearly indicated in the figure legends, which are currently missing this information.

We have revised all relevant figures (Figs. 4, 5, 6, 7, 8, 9, 10, 11, and Supplemental Figures) to clearly label the pink bars and markers as "Humans." We chose to label this data directly on the plots rather than in the legend box because the legend is used exclusively to differentiate DNN model characteristics (i.e. training strategy and datasets). We believe this direct labeling approach is the clearest way to present the information without causing confusion.

#R1_7 Missing Related Work on Human-Machine Comparison ---

7. Important studies on human-machine comparisons in other cognitive domains such as working memory and visual search are missing from the related work. Please consider including and discussing relevant works such as:
[a] https://arxiv.org/abs/2307.10768
[b] https://journals.plos.org/ploscompbiol/article?id=10.1371/journal.pcbi.1010654

We have revised the Introduction to cite the recommended papers on visual working memory [a] and visual search [b]. We also took the opportunity to add a relevant citation on material perception [c: 19-21] to further broaden the context:

(Introduction, p.2, ll.31-38)

These questions about the alignment of human and DNN strategies have been actively explored in other areas of vision research**.** While this is particularly notable in high-level tasks such as object recognition—a function often

associated with the ventral stream of the human brain—such comparisons now extend to a wide range of domains. These include mid-level perceptual judgments on material properties [19-21], as well as other fundamental cognitive functions like visual attention [22] and working memory [23]. In object recognition, early studies, such as those comparing CNN representations to activity in the primate inferotemporal cortex (IT) [24], …

#R1_8 Recent Advances in Depth Inference Models ---

8. Recent models like Neural Radiance Fields (NeRF) and 3D Gaussian Splatting can infer depth from images. Can the authors compare these models to human performance, or at least discuss their relevance in the related works section?

The primary reason we did not include these models in our direct comparison is the fundamental difference in the predominant task settings. Our study focuses on generalizable monocular depth estimation, where a model, once trained, infers a depth map from a single, unseen novel image. In contrast, standard NeRF and 3DGS are primarily designed for scene-specific reconstruction, requiring a set of multiple posed images of a particular scene to optimize a representation for that scene alone. This difference in input requirements and generalization capability makes a direct, fair comparison within our current framework challenging.

However, as you rightly suggest, an emerging and exciting line of research aims to generalize these 3D representations, enabling the inference of a NeRF or 3DGS model from a single image (e.g., Generalizable NeRFs). While these methods are highly relevant and promising, their primary objective is often novel view synthesis or full 3D shape recovery, rather than producing metrically accurate depth maps, which is the focus of our error analysis. The evaluation of their systematic biases in depth perception, in a manner comparable to the established depth estimation models we analyzed, remains an important avenue for future work.

To address your valuable point and to better contextualize our work, we will add a discussion of these models in the Discussion section of our revised manuscript.

(Limitations, p.23, ll.943-953)

Finally, we note key limitations in the scope of both the data and models analyzed, which point to important avenues for future work. Our study was based exclusively on indoor scenes from the NYU Depth V2 dataset. While this dataset is valuable for controlled comparisons, future studies should investigate a broader range of datasets, including outdoor scenes, to assess the generalizability of our findings and conclusions across diverse visual environments. Likewise, our investigation was largely restricted to discriminative models, aside from a few diffusion-based approaches. These generative approaches, along with others like generalizable NeRFs [84, 85], learn powerful priors to generate, not just estimate, plausible 3D structures. Whether this ability to synthesize scenes leads to a better alignment with human perceptual biases, compared to direct regression models, remains a critical question for future work.

#R1_9 Object Semantics in Depth Estimation ---

9. Object-level semantics can aid depth estimation—e.g., knowing the typical size of a sofa provides depth cues. Since the dataset includes ground truth segmentation masks, can the authors analyze category-specific depth estimation or explore whether there are any categorical biases?

We agree that analyzing category-specific depth estimation biases is a valuable addition. Following the reviewer's suggestion, we conducted a new analysis using the ground truth semantic segmentation masks from the NYU Depth V2 dataset. In summary, we calculated the per-category RMSE to investigate whether humans and DNNs share vulnerabilities to specific object types. Our analysis confirmed that they do, and furthermore, we found that DNNs with greater overall human-likeness also tend to share these specific category-level weaknesses more closely.

We have added a new paragraph detailing these findings and their implications to the manuscript, along with a new supplemental figure.

(Results > DNN depth judgments partially share human error patterns, pp.12-13, ll.470-494)

To dissect the nature of these shared human-DNN errors more deeply, we analyzed them at a semantic level. We first calculated the per-category RMSE for both humans and each DNN model using the pixel-wise semantic labels from the NYU Depth V2 dataset. Qualitatively, we observed that both humans and DNNs tend to exhibit larger errors for similar categories, particularly those with ambiguous depth cues like reflective surfaces (e.g., mirrors, windows) and ceilings (S3 FigA). This suggests that the shared errors are partly driven by difficulties with categories that are inherently ambiguous or lack strong geometric cues. However, a simple comparison of per-category errors could be confounded by depth: error magnitudes tend to increase with distance, and certain categories naturally appear at different depths. To isolate the effect of semantic category, we quantified this category-level similarity by computing the Pearson partial correlation between the human and DNN per-category RMSEs, while controlling for the average ground truth depth of each category. As shown in S3 FigB, many DNNs showed a positive partial correlation, confirming that they indeed share category-specific vulnerabilities with humans beyond what can be explained by depth alone. Finally, we explored whether this alignment at the category level is linked to a model's overall human-likeness. We compared the model rankings based on this new category-level similarity with the rankings from our main analysis of overall error pattern similarity (point-wise partial correlation). A Spearman rank correlation between these two ranking sets revealed a weak, positive relationship for both absolute data (ρ=0.313, 95%CI [-0.05, 0.6]) and scale-recovered data (ρ=0.223, 95%CI [-0.02, 0.44]) (S3 FigC). These results suggest a tendency for models with higher overall human similarity to also share the same category-level vulnerabilities, though the modest correlation indicates this link is not definitive and that overall similarity is shaped by more than just shared categorical biases.

(Supporting Information > S3 Fig)
Category-specific analysis for absolute and scale-recovered data. (A) Per-category RMSE analysis. The top plot shows the average ground truth distance for each semantic category. The plots below show the per-category RMSE for humans (purple squares) and DNNs (dots) for both absolute and scale-recovered data. DNN models are color-coded by their overall similarity to human judgments (measured by point-wise partial correlation). (B) Category-level human similarity for each DNN model. This metric measures the Pearson partial correlation between human and DNN per-category error patterns (from panel A) while controlling for per-category ground truth distance. (C) Relationships between category-level human similarity (from panel B) with overall point-wise human similarity.

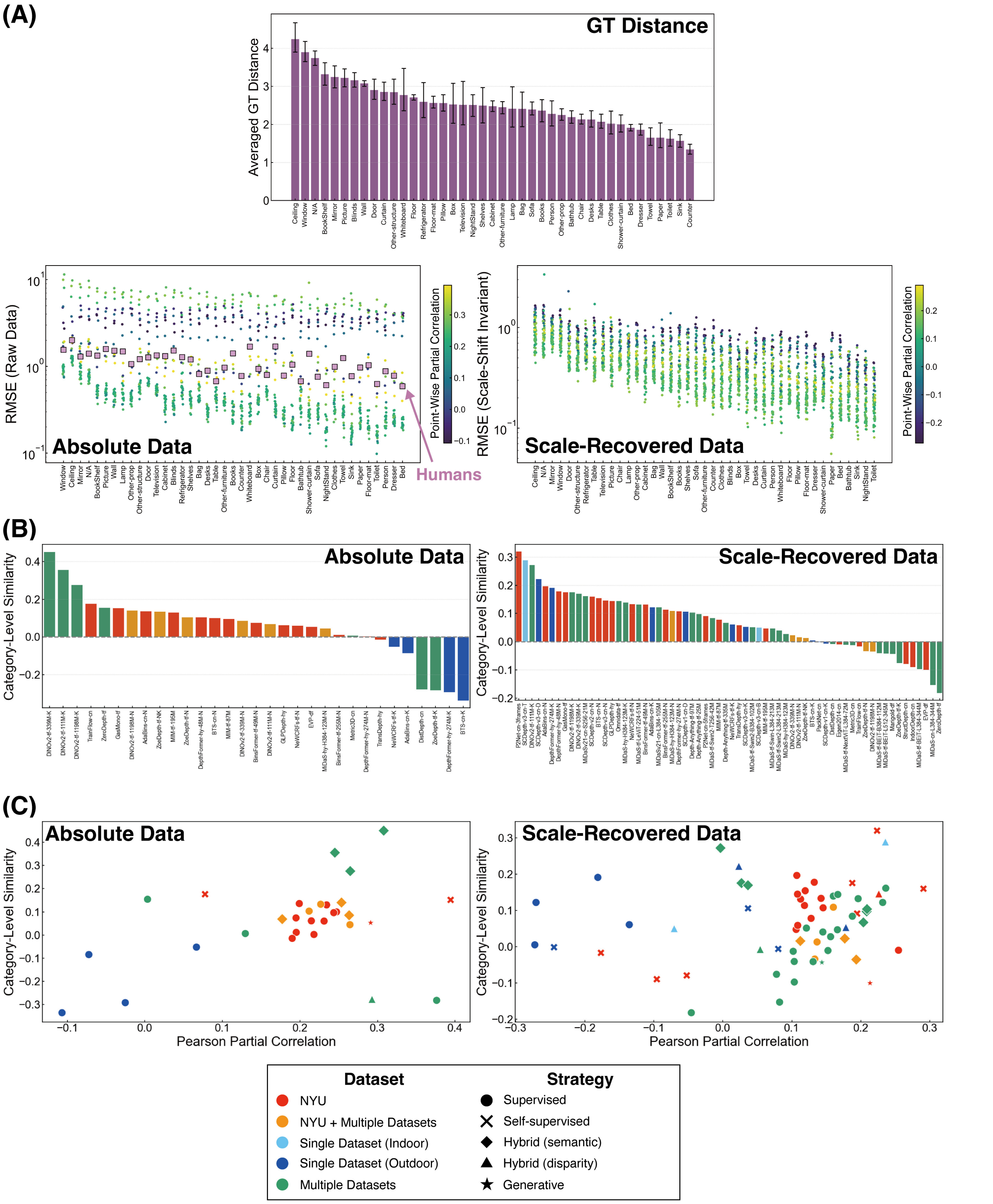

#R1_10 Data Availability Statement ---

10. The manuscript is missing a data availability statement. Please include one to support reproducibility.

We have uploaded full versions of analysis code and data to OSF repository (URL: https://osf.io/apkqn/). Our files include codes for model output, data collection, and data analysis. Additionally, we have uploaded a protocol describing the files and how to use them.

2. Responses to Reviewer #2

This work offers a novel perspective on comparing human depth perception with that of deep neural network (DNN)–based computer vision models. Through extensive statistical analyses and data comparisons, the authors explore both the similarities and differences in performance and attentional focus between humans and DNNs.

We deeply appreciate the reviewer's constructive comments. These comments will strengthen the rigorousness, significance, and value of our paper. The following is our responses to the points raised by Reviewer #2.

#R2_1---
However, the experimental setup lacks assessment and documentation of individual participants' habits and capabilities—for example, there is no record of ocular dominance (preferred or dominant eye). The authors attempt to mitigate individual behavioral biases by averaging across a random half-split of the human data, but the methodology and rationale for this "random half-split" procedure are not described in sufficient detail.

We acknowledge that factors like ocular dominance, which the reviewer pointed out, as well as other participant traits (e.g., visual acuity) and environmental specifics (e.g., precise viewing distance), were not recorded due to the nature of our crowdsourcing setup. However, given that our experiments were conducted under binocular viewing conditions (as clarified in our response to the subsequent comment) and involved a large number of participants (N=742), we believe that the influence of such individual traits on our main findings, which focus on general human perceptual tendencies, is likely minimal. To clarify this point for our readers, we have now added the following statement:

(Materials and methods > Procedures of collecting human dataset, p.25, ll.1041-1055)
Participants were recruited through a crowd-sourced online survey, with individuals ranging in age from their 20s to 40s (main dataset: 898 participants, supplemental dataset: 536 participants). All observations were conducted binocularly. Prior to the experiment, participants were instructed to disable any night mode or dark mode settings in their browsers and to view the display in full-screen mode. To ensure consistency in stimulus size across participants, they were asked to maintain an observation distance of 60 cm and to adjust a rectangle displayed on the screen until it matched the size of a credit card held in their hand. This process allowed for the calibration of the stimulus size based on the screen's pixel density. As a result, the image was presented at 9.6 cm x 7.2 cm, corresponding to a visual angle of 9.15 x 6.87. Our crowd-sourcing methodology precluded the collection of detailed participant information (e.g., visual acuity) and specifics of their viewing environment (e.g., precise viewing distance). While these uncontrolled factors could add noise to the data, their influence on our main findings is likely minimal, as our analyses are based on judgments averaged across a large participant pool.

The reviewer correctly points out that our primary focus is on the average characteristics of human depth perception. One of our intents with the random half-split procedure was to average out such individual-specific variations and random response noise to robustly extract the systematic error patterns shared across observers. We admit that the rationale and methodology for this procedure were not sufficiently detailed in the manuscript. To address this, we have added a more comprehensive explanation of the random half-split procedure—clarifying its purpose and steps:

(Results > Dataset collection for human depth judgments, p.5, ll.158-168)
To establish a human-level baseline or ``ceiling" for evaluating the performance of the DNNs, we needed to quantify the consistency of depth judgments across human observers. In the experiment, each participant responded to only a subset of the 654 images. To reliably measure how humans agree with each other across the full dataset, we adopted a random half-split procedure. This involves creating two ``pseudo-participant" datasets by randomly splitting and averaging the observers. This approach provides full data coverage and, by averaging out individual-specific response noise, allows for a more robust measurement of the error patterns that are systematically shared among human observers. By repeating this split 1,000 times, we obtained robust estimates and their 95% confidence intervals (CIs) for all subsequent human data analyses unless otherwise specified (see Materials and methods for detail).

Furthermore, to confirm the robustness of our approach, we have also performed supplementary analyses using individual-level responses (without averaging) and found that the main conclusions of this paper remain consistent.

For more details about this last point, please refer to our response to the second comment from Reviewer #1 (#R1_2a).

#R2_2---

In addition, the manuscript's stated motivation feels somewhat misaligned. The abstract frames the study as an investigation into distortions and biases in human 2D vision assisted by DNN, yet the Discussion shifts focus to the comparison of DNNs and human visual distortions. It might be clearer—and more impactful—to reposition the work as establishing a benchmark comparison between human observers and various state-of-the-art depth-estimation models.

As the reviewer pointed out, the framing of our motivation at the beginning of the Abstract could be better aligned with the discussion. Our core motivation, described in the Introduction, is that improving the human alignment of DNNs is important for both constructing functional models of the human visual system and designing artificial models with improved interpretability. However, this comparative aspect was not fully emphasized in the original Abstract, which may have given the impression of a study focused solely on elucidating human 3D vision.

Based on the reviewer's feedback, we have revised the first half of the Abstract to more clearly state our comparative approach. We have also reviewed the Authors Summary and Introduction to ensure this framing is consistent throughout the manuscript. The revised Abstract is as follows:

(Abstract)

Human depth perception from 2D images is systematically distorted, yet the nature of these distortions is not fully understood. By examining error patterns in depth estimation for both humans and deep neural networks (DNNs), which have shown remarkable abilities in monocular depth estimation, we can gain insights into constructing functional models of the human visual system and designing artificial models with improved interpretability. Here, we propose a comprehensive human-DNN comparison framework for a monocular depth judgment task. Using a novel human-annotated dataset of natural indoor scenes and a systematic analysis of absolute depth judgments, we investigate error patterns in both humans and DNNs. Employing exponential-affine fitting, we decompose depth estimation errors into depth compression, per-image affine transformations (including scaling, shearing, and translation), and residual errors. Our analysis reveals that human depth judgments exhibit systematic and consistent biases, including depth compression, a vertical bias (perceiving objects in the lower visual field as closer), and consistent per-image affine distortions across participants. Intriguingly, we find that DNNs with higher accuracy partially recapitulate these human biases, demonstrating greater similarity in affine parameters and residual error patterns. This suggests that these seemingly suboptimal human biases may reflect efficient, ecologically adapted strategies for depth inference from inherently ambiguous monocular images. However, while DNNs capture metric-level residual error patterns similar to humans, they fail to reproduce human-level accuracy in ordinal depth perception within the affine-invariant space. These findings underscore the importance of evaluating error patterns beyond raw accuracy, providing new insights into how humans and computational models resolve depth ambiguity. Our dataset and methodology provide a framework for evaluating the alignment between computational models and human perceptual biases, thereby advancing our understanding of visual space representation and guiding the development of models that more faithfully capture human depth perception.

#R2_3---

Moreover, while the paper employs monocular depth-estimation DNNs, it is not specified whether the human experiments were conducted monocularly or binocularly. This mismatch in input conditions could influence the findings and should be clarified or controlled.

Our human experiments were conducted under natural binocular viewing conditions. We agree that this choice warrants discussion. Under cue-poor conditions, binocular viewing can indeed provide a powerful flatness cue that attenuates perceived depth, as demonstrated by Koenderink et al. (1995).

However, we believe this effect is substantially mitigated in our study for two main reasons. First, our stimuli are cue-rich indoor scenes containing numerous objects with familiar sizes. This provides strong constraints on perceived depth, likely reducing the relative influence of the binocular flatness cue. Second, even if a systematic depth compression occurred, it would likely manifest as a global scaling effect. Our primary conclusions are based

on the patterns of systematic error (e.g., affine distortions) and ordinal judgments, which are robust to such global scaling.

We also argue that the role of binocular viewing in this context is more nuanced than it might first appear. While our task deviates from natural egocentric viewing (as we note in the first paragraph of our Limitation section), the human visual system is remarkably robust in interpreting depth from pictures under such conditions. This robustness has been attributed to a mechanism that uses binocular disparity to estimate the physical slant of the picture surface and compensate for the resulting geometric distortions (Vishwanath et al., 2005). From this perspective, binocular viewing in our experiment may have facilitated a more stable perception of the depicted scene, rather than simply acting as a conflicting flatness cue.

We have clarified the binocular viewing condition in the Materials and Methods section and have added the above discussion to the Limitation section to fully address this important point:

(Materials and methods > Procedures of collecting human dataset, p.25, ll.1041-1044)
Participants were recruited through a crowd-sourced online survey, with individuals ranging in age from their 20s to 40s (main dataset: 898 participants, supplemental dataset: 536 participants). All observations were conducted binocularly. Prior to the experiment, …

(Limitations, p.22, ll.908-919)
We acknowledge that our depth judgment task, while ecologically relevant, deviates from natural egocentric distance estimation, as participants judged depth from images on a flat screen. A key aspect of this deviation is that participants viewed the images binocularly, contrasting with the monocular input to the DNNs. While binocular disparity can indeed act as a flatness cue under sparse conditions [82], its role is not merely a source of cue conflict when viewing cue-rich pictures. Instead, the robustness of human vision in this context is thought to leverage this signal, as binocular disparity is used to estimate the picture's physical slant and actively compensate for geometric distortions [83]. This compensatory mechanism, supported by powerful pictorial cues such as familiar size, allows for a stable interpretation of the depicted scene. Thus, our findings likely capture fundamental aspects of this robust human system and its ability to effectively generalize depth perception from 2D representations.

Koenderink, J. J., van Doorn, A. J., & Kappers, A. M. (1995). Depth relief. Perception, 24(1), 115–126. https://doi.org/10.1068/p240115

#R2_4---
Finally, I recommend refining the layout for greater visual clarity: adjust figure placements and font sizes to ensure proper alignment and aesthetics, and relocate or resize some appendix images that currently interfere with the reference list.

We have adjusted the font sizes in the figures for better readability. Regarding their placement, we have separated all Figures (both main figures and supplemental figures) according to PLOS submission guidelines. Please check **#E4** for detail.

3. Additional Responses to Reviewers

#R3_1 Data Availability --

Reviewer #1: No: As far as I can tell, the zip folder only contains the post-processed results and the plots of the results. The models, the evaluation code, and the code for conducting human behavioral experiments are missing.
We have uploaded full versions of analysis code and data to OSF repository (URL: https://osf.io/apkqn/). Our files include codes for model output, data collection, and data analysis. Additionally, we have uploaded a protocol describing the files and how to use them.

#R3_2 Figures --

While revising your submission, please upload your figure files to the Preflight Analysis and Conversion Engine (PACE) digital diagnostic tool, https://pacev2.apexcovantage.com/. PACE helps ensure that figures meet PLOS requirements. To use PACE, you must first register as a user. Registration is free. Then, login and navigate to the UPLOAD tab, where you will find detailed instructions on how to use the tool. If you encounter any issues or have any questions when using PACE, please email PLOS at figures@plos.org. Please note that Supporting Information files do not need this step. If there are other versions of figure files still present in your submission file inventory at resubmission, please replace them with the PACE-processed versions.
We have checked all main figures in our manuscript using PACE format.

#R3_3 Reproductivity --

To enhance the reproducibility of your results, we recommend that authors of applicable studies deposit laboratory protocols in protocols.io, where a protocol can be assigned its own identifier (DOI) such that it can be cited independently in the future. Additionally, PLOS ONE offers an option to publish peer-reviewed clinical study protocols. Read more information on sharing protocols at https://plos.org/protocols?utm_medium=editorial-email&utm_source=authorletters&utm_campaign=protocols
We have uploaded full versions of analysis code and data to OSF repository (please check **#E_8**). Our protocol is clarified as the README.txt file, including requirements for reproduction and how to use our code.

4. Responses to Editors

#E_1---

1) Please ensure that the CRediT author contributions listed for every co-author are completed accurately and in full.

At this stage, the following Authors/Authors require contributions: Yuki Kubota, and Taiki Fukiage. Please ensure that the full contributions of each author are acknowledged in the "Add/Edit/Remove Authors" section of our submission form.

The list of CRediT author contributions may be found here:
https://journals.plos.org/ploscompbiol/s/authorship#loc-author-contributions

We have added Author Contributions with reference to CRediT format.

#E_2---

2) We ask that a manuscript source file is provided at Revision. Please upload your manuscript file as a .doc, .docx, .rtf or .tex. If you are providing a .tex file, please upload it under the item type u2018LaTeX Source Fileu2019 and leave your .pdf version as the item type u2018Manuscriptu2019.

We have uploaded LaTeX source files.

#E_3---

3) Thank you for including an Ethics Statement for your study. Please include:

i) A statement that formal consent was obtained (must state whether verbal/written) OR the reason consent was not obtained (e.g. anonymity). NOTE: If child participants, the statement must declare that formal consent was obtained from the parent/guardian.].

Before the start of the experiment, we collected electronic informed consent from participants. To clarify this in the manuscript, we have added the following description to the Ethical statement

(Ethical Statement > p.23, ll.974-979)

All psychophysical experiments were conducted in accordance with the Declaration of Helsinki, and their experimental designs were approved by the Institutional Review Board of NTT Communication Science Laboratories. For the online experiments, the participants provided their electronic informed consent to participate in this study. Only those who fully understood the content and purpose of the experiment and provided their consent online participated in the experiment.

#E_4---

4) Please upload all main figures as separate Figure files in .tif or .eps format. For more information about how to convert and format your figure files please see our guidelines:
https://journals.plos.org/ploscompbiol/s/figures

We have uploaded all main figures as separate files with TIF format.

#E_5---

5) We have noticed that you have uploaded Supporting Information files, but you have not included a complete list of legends. Please add a full list of legends for your Supporting Information files (analysis.zip) after the references list.

We have updated a full list of Supporting Information files. As mentioned in **#E_8**, we uploaded analysis code and data in OSF repository.

#E_6---

6) We notice that your supplementary Figures, and information are included in the manuscript file. Please remove them and upload them with the file type 'Supporting Information'. Please ensure that each Supporting Information file has a legend listed in the manuscript after the references list.

We have uploaded Supporting Information, including S1 to S7 Figs (TIF), S1 Appendix (PDF), and S1 Table (CSV), as separate files from the manuscript.

#E_7---

7) Some material included in your submission may be copyrighted. According to PLOSu2019s copyright policy, authors who use figures or other material (e.g., graphics, clipart, maps) from another author or copyright holder must demonstrate or obtain permission to publish this material under the Creative Commons Attribution 4.0 International (CC BY 4.0) License used by PLOS journals. Please closely review the details of PLOSu2019s copyright requirements here: PLOS Licenses and Copyright. If you need to request permissions from a copyright holder, you may use PLOS's Copyright Content Permission form.
Please respond directly to this email and provide any known details concerning your material's license terms and permissions required for reuse, even if you have not yet obtained copyright permissions or are unsure of your material's copyright compatibility. Once you have responded and addressed all other outstanding technical requirements, you may resubmit your manuscript within Editorial Manager.
Potential Copyright Issues:
i) Please confirm (a) that you are the photographer of 1, and 12, or (b) provide written permission from the photographer to publish the photo(s) under our CC BY 4.0 license.

We consulted with the editor in advance. Since we were unable to obtain a written permission form from the original copyright holder, we used ChatGPT to generate images for illustration. We have confirmed "Terms of Use" of ChatGPT in accordance with the editor's comments and replaced Figures 1 and 12 with generated images.

ChatGPT's Terms of Use: https://openai.com/policies/row-terms-of-use/

Additionally, we have clearly denote that generative AI was used in the creation of images within the figure legends.
"The image was generated by ChatGPT (GPT-4o, 2025) to avoid potential copyright issues and is intended for illustrative purposes only."

#E_8---

8) Thank you for stating "Human and model data, and data analysis code are available on Github (https://github.com/yuki-kubota-95/human-like-MDE)." This link reaches a 404 error page. Please amend this to a new link or provide further details to locate the data.
We have uploaded data related to our work to the OSF repository. The link is https://osf.io/apkqn/.
